# Estimating the Effects of a Hurricane on Carbon Storage in Mangrove Wetlands in Southwest Florida

**DOI:** 10.3390/plants10081749

**Published:** 2021-08-23

**Authors:** Lauren N. Griffiths, William J. Mitsch

**Affiliations:** 1 Everglades Wetland Research Park, The Water School, Florida Gulf Coast University, 4940 Bayshore Drive, Naples, FL 34112, USA; wmitsch@fgcu.edu; 2 School of Geosciences, University of South Florida, 4202 E. Fowler Avenue, Tampa, FL 33620, USA

**Keywords:** mangrove swamps, hurricane, carbon storage, subtropical Florida, hurricane Irma, climate change

## Abstract

Tropical and subtropical mangrove swamps, under normal conditions, can sequester large amounts of carbon in their soils but as coastal wetlands, they are prone to hurricane disturbances. This study adds to the understanding of carbon storage capabilities of mangrove wetlands and explores how these capacities might change within the scope of a changing storm climate. In September 2017, Naples Bay, FL, USA (28°5′ N, 81°47′ W) encountered a direct hit from hurricane Irma, a Saffir–Simpson category 3 storm. By comparing carbon storage, forest community structure, and aboveground productivity collected in 2013 and in 2019, we estimated the effects of hurricane Irma on mangrove functions. Aboveground biomass increased during the study period at a rate of approximately 0.72 kg m^−2^ yr^−1^, significantly less than the average found in undisturbed mangrove forests. Soil carbon storage decreased at all study sites. On average, 2.7 kg-C m^−2^ was lost in the top 20 cm between sample collections. Carbon loss in belowground pools could point to a feedback of mangrove swamps on climate change as they lose their ability to store carbon and increase net atmospheric carbon. Nevertheless, mangrove swamps remain resilient to tropical storms in the long term and can recover their carbon storage capacity in the years following a storm.

## 1. Introduction

Excess carbon dioxide in the atmosphere is a major global concern, causing increased atmospheric temperatures, rising sea levels, and theoretically more frequent intense storms, among other widespread issues [1,2,3,4]. The world’s wetlands have been estimated to sequester as much as 1 Pg-C yr^−1^, which was, at the time, approximately 10% of the total carbon emitted from burning fossil fuels [5,6]. Mangrove ecosystems sequester large amounts of carbon because of a combination of their carbon-rich productive vegetation and high sedimentation rates [6,7,8,9,10]. Mangroves are also unique because, in normal conditions, they rarely emit the greenhouse gas methane unlike freshwater wetlands [7,11], making them important atmospheric carbon sinks. Mangroves also protect coastlines from hurricane and tropical storm damage [12,13,14]. Yet, with climate change effects such as sea level rise and potential increased storm intensity coupled with human development, mangroves and their large carbon pools are at risk [6,10].

Of the large carbon stores in mangrove swamps, organic carbon is the main fraction, making up as much as 89–99% of the total belowground carbon stock [10]. Organic carbon inputs to the system can be from allochthonous sources such as precipitation and ocean or estuarine-derived phytoplankton, seagrasses, or microphytobenthos. However, the main portion of organic matter is autochthonous from mangrove litter [15,16]. Terrigenous and siliciclastic estuarine and oceanic influxes dominate the inorganic carbon inputs to Naples Bay mangroves [17]. The main pathways for organic carbon decomposition are aerobic respiration and anaerobic sulfate reduction [6,15,18]. Under oxidized conditions, aerobic respiration leads to organic carbon transformation to carbon dioxide [15,18]. In anoxic conditions, methanogenesis is low because sulfate-reducing microorganisms dominate, leading to organic carbon transformations and carbon dioxide releases rather than methane emissions [6,15,18,19].

The effect that increased hurricane activity might have on mangrove carbon storage is poorly understood. Smoak et al. [20] suggest that storms may play a significant role in maintaining long-term carbon storage of mangrove wetlands by depositing a layer of inorganic carbon-rich sediment on top of the organic soil, whereas other studies suggest that intense storms may lead to peat collapse and subsequent carbon loss [21,22]. The result may be dictated by the mangrove mortality rate and subsequent regeneration and recovery of the ecosystem after a hurricane where mass mortality may lead to peat collapse and carbon loss, whereas, if mangroves remain alive, peat collapse is less likely to occur and increased sediment and carbon storage may occur [20,21]. This study will explore whether a Saffir–Simpson category 3 hurricane in subtropical south Florida in 2017 increased, decreased, or did not affect overall carbon storage of mangroves at locations sampled before and after the hurricane. If large storms cause a release of carbon, this may indicate that there is a positive feedback on climate change when hurricanes and tropical and extratropical storms temporarily disturb mangrove aboveground biomass, causing erosion and loss of previously sequestered soil. These biomass and soil changes could then reduce the capacity of the mangroves to store and sequester carbon which, in turn, would increase greenhouse gases in the atmosphere. Alternately, inorganic carbon-rich storm deposits could be vital to long-term belowground carbon storage by burying organic carbon and protecting it from future erosion or oxidation [20]. In this case, storm surges deposit inorganic carbon-rich sediments over organic soils, and coastal sites are more likely to have large storm deposits since they are closer to the source of inorganic sediments and are impacted more by storm surge compared to inland sites [20].

The goal of this study is to gain a better understanding of soil carbon dynamics in mangroves and how this is affected by hurricane and tropical storm activity. This study analyzes how soil carbon pools in both hydrologically disturbed and undisturbed mangrove tidal creeks are changed by hurricanes by comparing soil cores collected before (2013–2014) and after (2019) hurricane Irma.

This study tests the following hypotheses on two tidal creeks in southwest Florida by comparing pre- and post-hurricane collected soil cores.

1. Samples taken post-hurricane will have a thicker layer of inorganic carbon-rich, organic carbon-poor sediments compared to pre-hurricane samples.
2. After a hurricane, fringe mangrove sites will have more sediment accumulation, but less organic carbon accretion compared to the further inland, riverine mangrove sites.
3. Hurricanes and large storms will decrease carbon storage in mangrove swamps near open water.

## 2. Results

### 2.1. Mangrove Forest Community Structure

The dominant species at all sites was *R. mangle*, which ranged from 54 to 100% of stem counts (Table 1). The other species identified in this study was *L. racemosa*. No *A. germinans* was present in the 2019 study plots, although a single individual was present at one site in 2014. *R. mangle* tree density was greatest in the fringe site in the reference creek (0.76 stems m^−2^) and was greater than the 2014 density at that site (0.39 stems m^−2^). *R. mangle* tree density increased at the two fringe sites from 0.46 and 0.39 stems m^−2^ in 2014 to 0.48 and 0.76 stems m^−2^ in 2019 at the disturbed and reference creeks, respectively. *R. mangle* tree density decreased, however, at the two riverine sites from 0.77 and 0.51 stems m^−2^ in 2014 to 0.56 and 0.48 stems m^−2^ in 2019 at the disturbed and reference creeks, respectively.

**Figure 1:**
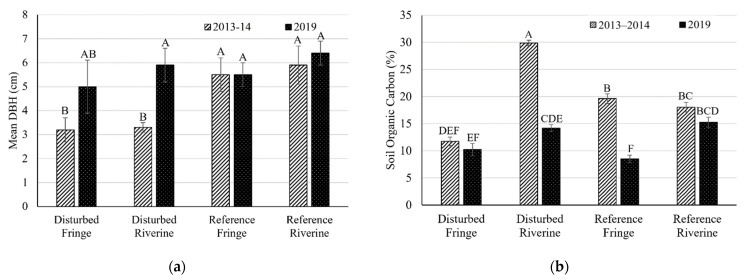
(**a**) Mean diameter at breast height (DBH) of *Rhizophera mangle* and (**b**) soil organic carbon at the fringe and riverine settings in the disturbed and reference creeks in the period 2013–2014 [10] and 2019 (this study). Error bars represent ± standard error. Similarity letters represent the results of a Tukey HSD test.

Average diameter at breast height (DBH) increased over time for *R. mangle* at all sites except for the reference fringe site where average DBH remained the same (Table 1; Figure 1a). There was a statistically significant increase in *R. mangle* DBH at the riverine setting in the disturbed creek from 3.3 ± 0.2 cm in 2014 to 5.9 ± 0.7 cm in 2019 (*t*-test, *p* < 0.01). Where *L. racemosa* was present, mean DBH decreased between studies at all sites (Table 1).

Estimated aboveground biomass in the period 2013–2014 averages 9.78 ± 1.55 kg m^−2^, whereas 2019 biomass averages 13.5 ± 2.48 kg m^−2^ (Table 1). In 2019, the site with the greatest biomass was the disturbed creek riverine site with 18.77 ± 0.15 kg m^−2^ which increased from 8.43 ± 0.03 kg m^−2^ in the initial study, followed by the reference creek fringe site with 16.69 ± 0.21 kg m^−2^, increased from 13.08 ± 0.09 kg m^−2^. The disturbed creek fringe site had 6.14 ± 0.04 kg m^−2^ in the period 2013–2014 and 9.18 ± 0.27 kg m^−2^ in 2019. The reference creek, riverine site had the only noted decrease in aboveground biomass with 11.49 ± 0.05 kg m^−2^ initially and 9.34 ± 0.15 kg m^−2^ in this study. This is the only site that had *L. racemosa* present in the period 2013–2014, but none present in 2019. All creeks and sites had an increase in aboveground biomass of *R. mangle*, whereas *A. germinans* was present at one site in the period 2013–2014, but was not present at any sites in 2019 and *L. racemosa* decreased in aboveground biomass at the disturbed fringe site and the reference riverine site.

### 2.2. Soil Bulk Density

**Figure 2:**
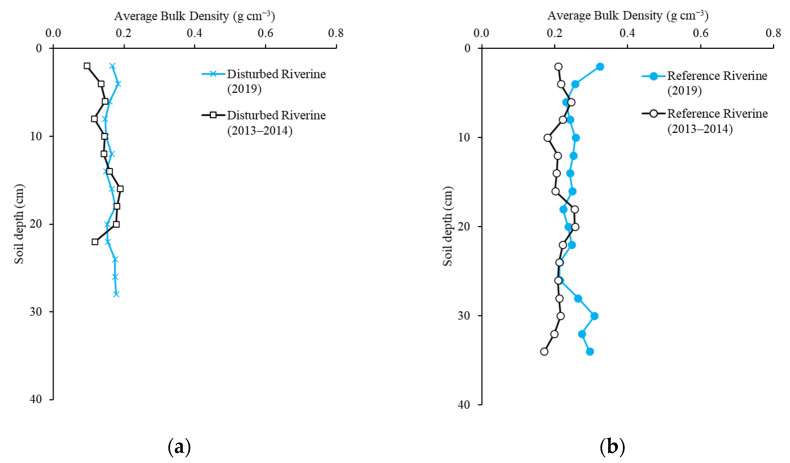

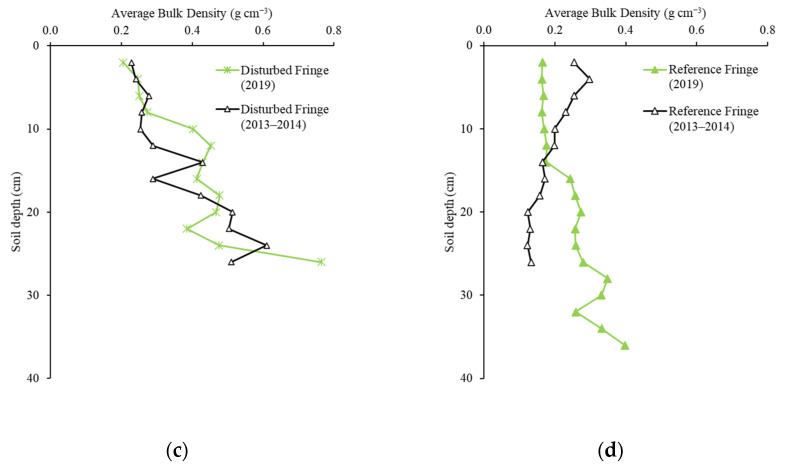
Average bulk density of soil in the period 2013–2014 and 2019 at each 2 cm of depth in (**a**) the disturbed riverine site; (**b**) the reference riverine site; (**c**) the disturbed fringe site; and (**d**) the reference riverine site.

Average bulk density ranged from 0.15 to 0.76 g cm^−3^ across all sites and depths (Figure 2). Mean bulk density in this study was 0.26 ± 0.01 g cm^−3^. There was no statistical difference in bulk density in 2019 compared to 2013–2014 with 0.23 ± 0.02 g cm^−3^ across all sites and depths sampled (repeated-measures ANOVA, *p* = 0.14) [10]. Bulk density at both sites in the hydrologically disturbed creek (Figure 2a,c) were similar to those recorded in the period 2013–2014 with bulk density values greater at all depths of the fringe mangroves (2019: 0.39 ± 0.03 g cm^−3^; 2013–2014: 0.37 ± 0.04 g cm^−3^) than those in the riverine mangroves (2019: 0.16 ± 0.01 g cm^−3^; 2013–2014: 0.15 ± 0.01 g cm^−3^) [10]. Bulk density at the riverine site in the reference creek was statistically different between 2013–2014 and 2019 with 0.21 ± 0.01 g cm^−3^ and 0.25 ± 0.01 g cm^−3^, respectively (Figure 2b; repeated-measures ANOVA, *p* < 0.01). The bulk density was statistically different at the reference creek fringe site as well, with values of 0.19 ± 0.02 g cm^−3^ in the period 2013–2014 and measures of 0.24 ± 0.01 g cm^−3^ in 2019 (Figure 2d; repeated-measures ANOVA, *p* = 0.02).

### 2.3. Soil Carbon Profile

**Figure 3:**
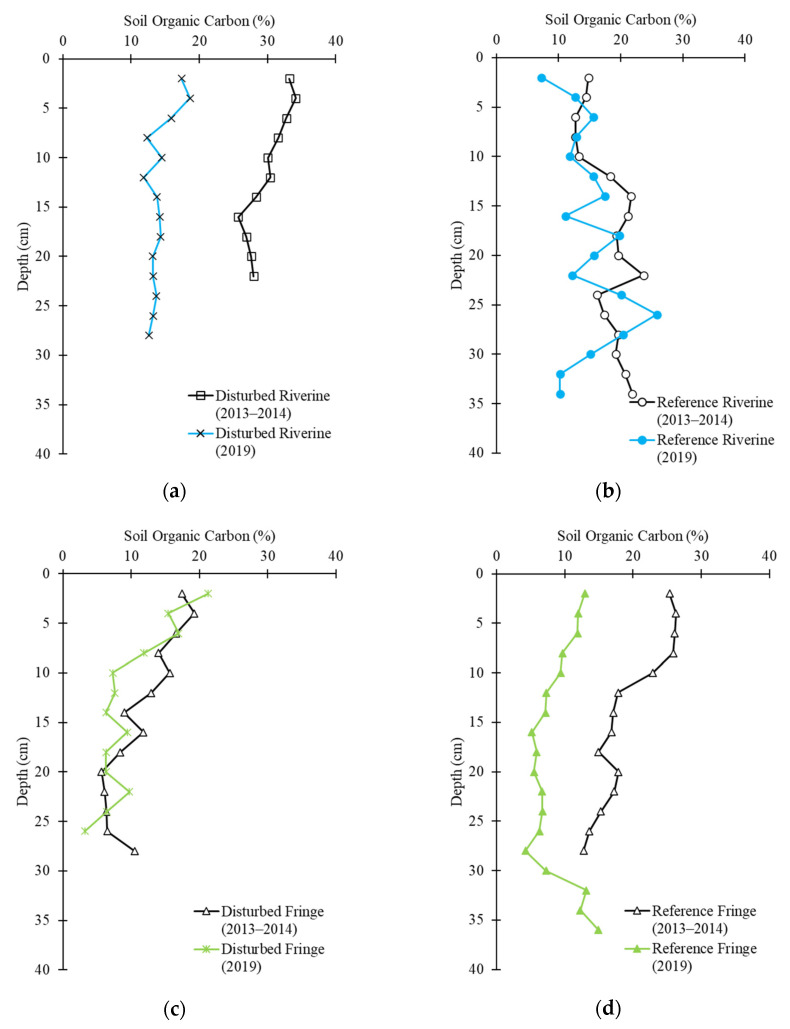
Soil organic carbon concentration in the period 2013–2014 and 2019 at each 2 cm soil depth in (**a**) the disturbed riverine site; (**b**) the reference riverine site; (**c**) the disturbed fringe site; and (**d**) the reference riverine site.

**Figure 4:**
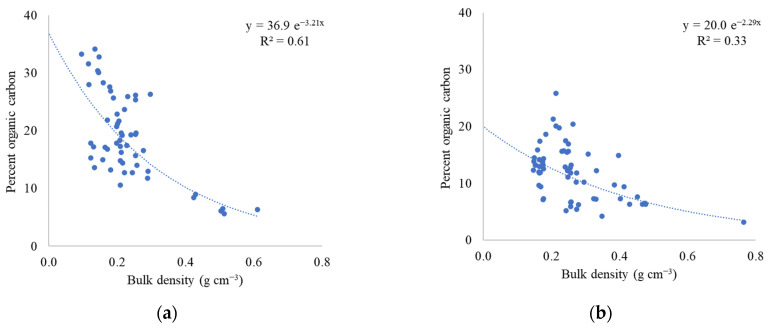
Regression predicting percent organic carbon based on soil bulk density in (**a**) 2013–2014 and (**b**) 2019 from all sites studied.

Belowground organic carbon (OC) content ranged from 32 to 258 g-C kg^−1^ in the different mangrove hydrogeomorphic settings at different depths and the mean was 121 ± 6 g-C kg^−1^. This is statistically lower than the average carbon content in the period 2013–2014 of 191 ± 10 g-C kg^−1^ (Kruskal–Wallis, *p* < 0.01) [10]. Mean OC content in the reference creek was 186 ± 7 g-C kg^−1^ in the period 2013–2014 [10] and 118 ± 8 g-C kg^−1^ in 2019. Mean OC in the disturbed creek were 196 ± 20 g-C kg^−1^ in the period 2013–2014 [10] and 121 ± 8 g-C kg^−1^ in 2019. The highest OC was 258 g-C kg^−1^ at 26 cm depth in the reference creek riverine site and the lowest OC was 32 g-C kg^−1^ at 26 cm depth in the disturbed creek fringe site. There was no statistical difference between OC at different depths (repeated-measures ANOVA; *p* = 0.99). OC content was statistically greater at the two riverine sites compared to the two fringe sites (Figure 3; Kruskal–Wallis; *p* < 0.01). OC content decreased at all sites from 2013–2014 to 2019 (Figure 1b). Organic carbon content from both studies was negatively correlated to the bulk density of a specific sample (Figure 4).

**Figure 5:**
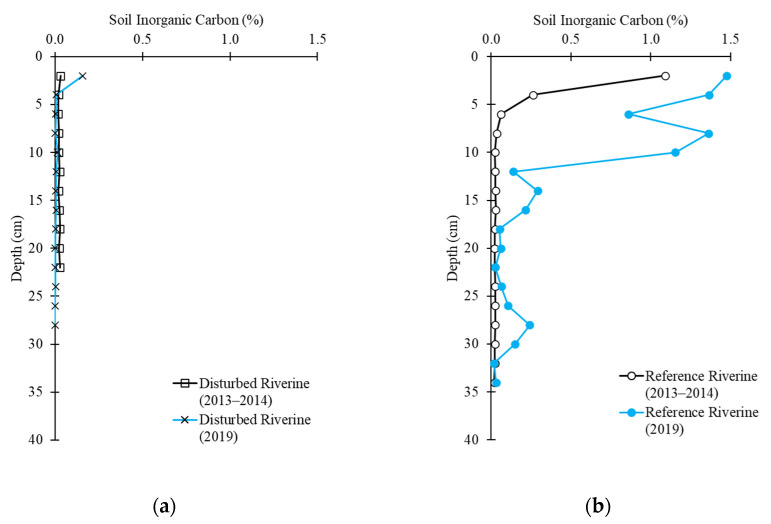

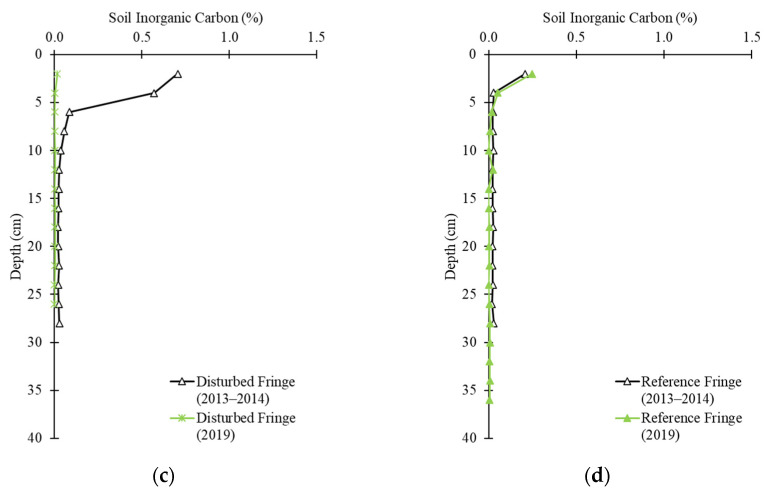
Soil inorganic carbon concentration in the period 2013–2014 and 2019 at each 2 cm of depth in (**a**) the disturbed riverine site; (**b**) the reference riverine site; (**c**) the disturbed fringe site; and (**d**) the reference fringe site.

At all study sites, both pre- and post-hurricane, organic carbon made up 99% of the total carbon fractions, inorganic carbon made up only 1% of the total carbon in the soil. Inorganic carbon content ranged from 0 to 15 g-C kg^−1^ across all sites and depths (Figure 5). Inorganic carbon content was the greatest at the reference creek riverine site. Higher inorganic carbon (IC) content was identified in the top 2 cm in each site, but statistical difference was only found at the disturbed creek riverine site (1.5 g-C kg^−1^ of IC at 2 cm; repeated-measures ANOVA, *p* < 0.01) and the reference creek fringe site (2.5 g-C kg^−1^ of IC at 2 cm; repeated-measures ANOVA, *p* < 0.01).

### 2.4. Soil Carbon Change

**Figure 6:**
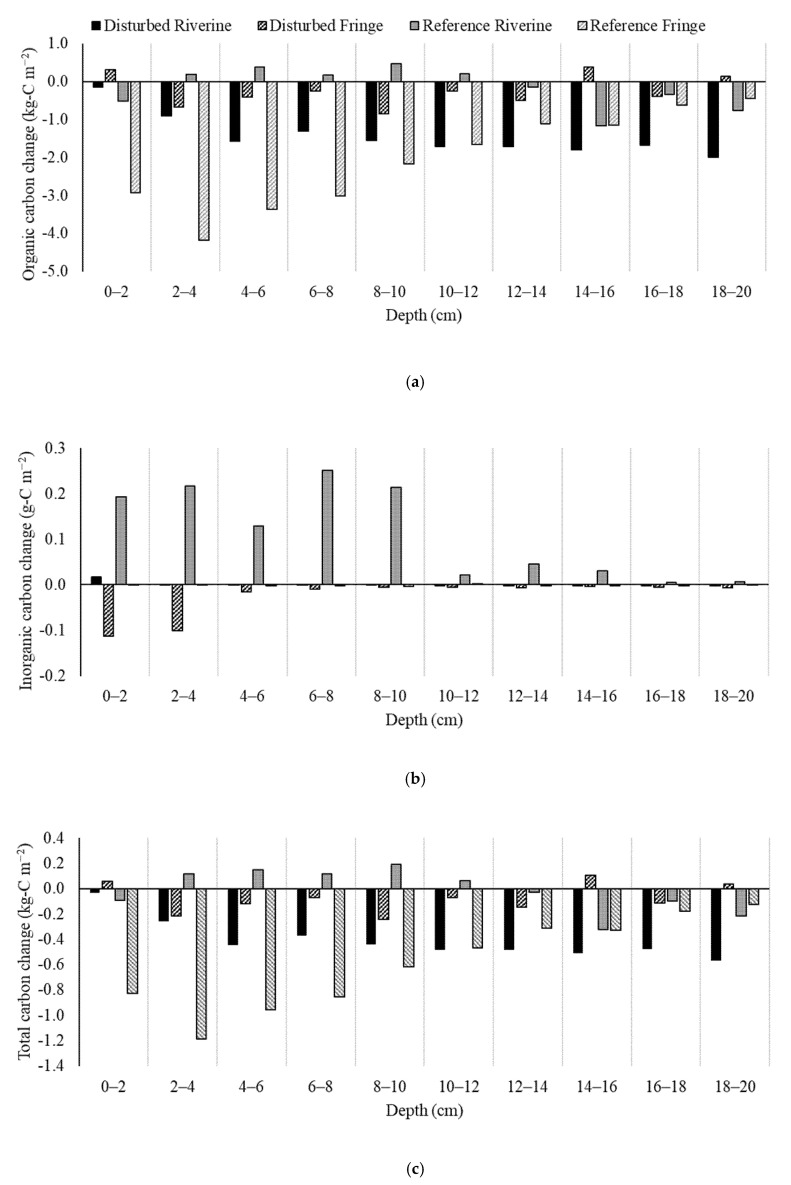
Change in (**a**) organic carbon, (**b**) inorganic carbon, and (**c**) total carbon at each 2 cm of depth of the soil core (70.5 cm^3^) in the two hydrogeomorphic settings (fringe and riverine) in the disturbed and reference creeks between 2013–2014 and 2019.

In the top 20 cm of the soil, there has been an approximately 2.71 kg-C m^−2^ decrease in total carbon from 2013–2014 to 2019 (Table 2; Figure 6). The greatest decrease in total carbon occurred at the reference creek fringe site where there was a difference of −5.86 kg-C m^−2^ in the top 20 cm of the soil from 2013–2014 to 2019. The reference creek riverine site had the least amount of carbon change with only −0.12 kg-C m^−2^, followed by the disturbed creek fringe site with −0.78 kg-C m^−2^ in the top 20 cm. The top 20 cm of soil at the disturbed creek riverine site decreased by 4.07 kg-C m^−2^.

Organic carbon is the main fraction of carbon loss at all sites with approximately 2.77 kg-C m^−2^ lost between sampling periods in the top 20 cm (Table 2; Figure 6). Organic carbon loss ranged from 0.43 to 5.86 kg-C m^−2^ in the top 20 cm of soil. The average inorganic carbon across all sites increased by 0.058 kg-C m^−2^, but this average is skewed by a large increase at the reference creek riverine site which gained 0.32 kg-C m^−2^ of inorganic carbon in the top 20 cm. The other three sites had varying inorganic carbon changes with 0.00021 kg-C m^−2^ gained at the disturbed riverine site (mostly in the top 2 cm), 0.077 kg-C m^−2^ lost in the disturbed creek fringe site, and 0.005 kg-C m^−2^ lost in the reference fringe site. The two riverine sites gained 0.00021 to 0.315 kg-C m^−2^ while the two fringe sites lost 0.005 to 0.022 kg-C m^−2^ of inorganic carbon. The disturbed creek gained less inorganic carbon and lost more inorganic carbon than the reference creek.

### 2.5. Total Carbon Stock

**Figure 7:**
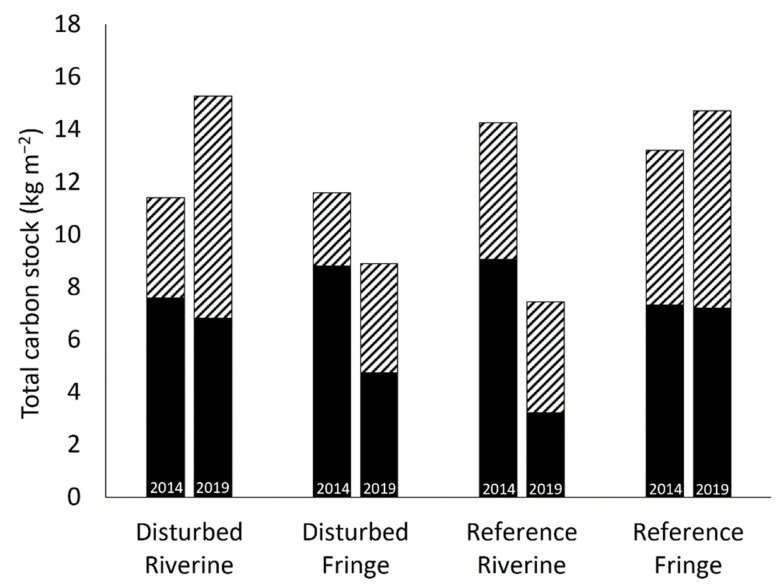
Total carbon stock in aboveground (grey stripes) and belowground (solid black) stores in the two hydrogeomorphic settings (fringe and riverine) in the hydrologically disturbed and reference creeks in the period 2013–2014 and 2019.

The total carbon stock at each site, as a function of above- and belowground carbon storage shows the total carbon stored in the period 2013–2014 and 2019 (Figure 7). In the hydrologically disturbed creek, the fringe site had a large decrease in belowground carbon (8.89 to 4.76 kg-C m^−2^) and a small increase in aboveground carbon (2.76 to 4.13 kg-C m^−2^), leading to an overall decrease in total carbon stored at the site of 2.70 kg-C m^−2^. At the riverine site in the hydrologically disturbed creek, there was an overall increase in carbon stored of 3.87 kg-C m^−2^ as a result of a small decrease in belowground carbon (7.60 to 6.82 kg-C m^−2^) but a large increase in aboveground carbon (3.79 to 8.44 kg-C m^−1^). In the reference creek, the fringe site had an overall increase of 1.50 kg-C m^−2^ carbon stored at the site as a result of an almost insignificant change in belowground carbon (7.32 to 7.21 kg-C m^−2^), but an increase in aboveground carbon (5.89 to 7.51 kg-C m^−2^). The reference site had an overall decrease in carbon of 6.83 kg-C m^−2^ lost from 2013–2014 to 2019 at the riverine site due to decreases in both above- (5.17 to 4.20 kg-C m^−2^) and belowground (9.08 to 3.22 kg-C m^−2^) carbon storage.

### 2.6. Carbon Sequestration

Throughout the entire core, the sedimentation rate was higher at the reference site than the disturbed site (Table 3). The disturbed fringe site had a sedimentation rate of 1.78 mm yr^−1^, the disturbed riverine site had a sediment accretion rate of 1.91 mm yr^−1^, the reference fringe site had a sediment accretion rate of 3.69 mm yr^−1^, and the reference riverine site had a sediment accretion rate of 4.02 mm yr^−1^. On average, carbon sequestration was greatest at the reference riverine site (154.2 g-C m^−2^ yr^−1^), followed by the reference fringe (70.0 g-C m^−2^ yr^−1^), the disturbed fringe (62.6 g-C m^−2^ yr^−1^), and the disturbed riverine with the lowest carbon sequestration rate (44.9 g-C m^−2^ yr^−1^). There was no statistical change in carbon sequestration rates throughout the cores at each site.

## 3. Discussion

### 3.1. Hurricane Effects on Mangrove Community Structure

Sites which had a loss of *R. mangle* tree density had an increase in mean DBH. The one site that had an increase in *R. mangle* tree density had a decrease in mean DBH. This suggests that hurricane Irma affected three out of the four sites by decreasing the number of individual trees, but the trees that did survive, continued to grow in size, thus not affecting (and in fact increasing) overall aboveground biomass. Total biomass increased by approximately 2.71 kg m^−2^ in the five years between studies, making the annual aboveground biomass growth rate approximately 0.72 kg m^−2^ yr^−1^. What likely occurred in this mangrove wetland is a result of small gaps in the mangrove canopy, which stimulates growth and recolonization from mangrove seedlings or expansion of surviving trees [23]. Large, landscape scale gaps, on the other hand are usually detrimental to mangrove regrowth because of decreasing redox and increasing sulfide levels when there are large mangrove mortalities [23]. Throughout southern Florida, mangroves were damaged initially, regrowth occurred, and delayed mortality was cited 3–9 months after the hurricane had passed [24], during which time adequate regrowth would have prevented fatal levels of redox and sulfides.

Aboveground biomass production of stems was similar in the Biscayne National Park site in Florida compared to our study, averaging 0.67 ± 0.1 kg m^−2^ yr^−1^ [25], the Naples Bay fringe mangroves were estimated to have stem productivity of 0.72 kg m^−2^ yr^−1^ post-hurricane, indicating that hurricane Irma may not have had a significant effect on aboveground productivity long-term post-hurricane. Although immediately after hurricane Irma there were visual signs of aboveground vegetation damage, in the two years since the hurricane hit Naples Bay, the mangroves have been able to significantly recover and have greater biomass than was present in the period 2013–2014.

### 3.2. Soil Carbon Change

The hypothesis that post-hurricane samples would have a layer of inorganic carbon-rich, organic carbon-poor sediments, was not supported by carbon content in the upper layers of the soil at most sites. Although soil organic carbon, on average, decreases pre- to post-hurricane (Table 2), inorganic carbon does not significantly differ pre- to post-hurricane. At the riverine site of the reference creek, there was a significant increase in inorganic carbon in the top 16 cm of the soil core in 2019 compared to 2013. Smoak et al. [20] noted a trend in the Everglades National Park, approximately 100 km southeast of Naples Bay, where storms deposited carbonate-rich, organic carbon-poor sediments on top of the organic rich mangrove soils which was only seen at this one site in this study. Carbon dynamics were different from this study because of the hydrologic and geologic differences between these sites. Our sites in Naples Bay are within a bay and not directly on the coast, limiting carbonate depositions to this site and relying on terrigenous inorganic carbon and siliciclastic inputs, whereas the Everglades sites have direct access to coastal inputs and are carbonate dominant [17]. Terrigenous-dominated mangrove swamps will likely respond similar to this study with no added inorganic carbon layer of sediments, whereas coastal-dominated systems have inorganic carbon deposits that preserve long-term carbon sequestration [20].

The hypotheses that organic carbon and total carbon storage would decrease as a result of a hurricane were supported by this study. Soil carbon content decreased across all sites in the top 20 cm from 2013–2014 to 2019. It is not likely that erosion or storm deposits account for these changes since dated soils do not show evidence of a change in sedimentation or carbon sequestration rates in the top layers of the soil profile at most of the sites. There are two other possible explanations for this decrease in carbon at the sites. The first possibility is peat collapse. The two sites at the reference creek, increased soil bulk density and decreased total carbon storage. Increased bulk density in surface or shallow subsurface of the soil is often a good indicator of peat collapse [26]. It is well documented that peat collapse can occur in mangroves after hurricanes and can continue at rates up to 7–11 mm yr^−1^ for at least 8 years after the hurricane if roots fail to regrow in the area [22]. Since aboveground productivity was not significantly impacted in Naples Bay by hurricane Irma, peat collapse did not likely significantly affect these sites in the long term. At all sites, 2 years post-hurricane, aboveground biomass has increased, suggesting that there will be little long-term effects on overall carbon storage at these sites.

The other likely explanation of organic carbon decrease is that carbon was allocated from roots to aboveground growth. Since belowground biomass is not significantly affected by hurricanes, roots do not require stored carbon, whereas storm-affected trees require belowground stored carbon to increase aboveground biomass regeneration [27]. This is further supported by the aboveground biomass data, which show that the two sites with the highest amount of aboveground biomass (the reference fringe with 16.7 kg m^−2^ and the disturbed riverine with 18.8 kg m^−2^) have the greatest belowground carbon loss (5860 and 4071 g-C m^−2^, respectively). Mangroves with added nutrients allocate shifts in growth from roots to shoots, thus increasing aboveground biomass production [28]. Hurricanes are important in their addition of phosphorus to mangrove sediments [29]; thus, after hurricane Irma, the mangroves in Naples Bay likely increased aboveground production, which may have caused a significant decrease in carbon stocks belowground. This is further illustrated where the site with the least amount of carbon loss (reference riverine with 118 g-C m^−2^ lost) is the only site where total aboveground biomass decreased pre- to post-hurricane (−2.15 kg m^−2^ from 2013–2014 to 2019). This is not only likely due to the lack of carbon allocation to aboveground production, but also, this site had many downed trees which caused regrowth to be more difficult.

### 3.3. Total Carbon Stock

Total carbon stock increased at the two sites (the disturbed riverine and the reference fringe) where belowground carbon did not significantly decrease. This suggests that belowground carbon stocks regulate overall above- and belowground carbon storage. When belowground carbon decreases (the disturbed fringe and reference riverine), aboveground carbon stores act to buffer the overall carbon loss. At the two sites, belowground carbon decreased by 4–6 kg-C m^−2^, but at the disturbed fringe site, aboveground carbon increased by 1.4 kg-C m^−2^, whereas carbon loss was compounded at the reference riverine site by an additional loss of almost 1 kg-C m^−2^ in aboveground carbon. This highlights the importance of aboveground biomass regrowth after a hurricane to help buffer carbon loss in belowground storage.

### 3.4. Carbon Sequestration

Sediment accretion and carbon sequestration rates were greater at the reference sites compared to the hydrologically disturbed sites in both 2013–2014 and 2019 [10]. This suggests that freshwater inputs are a significant source of sediments to these mangrove wetlands and when upstream hydrology is disturbed, the sediments and organic matter are not stored within mangrove wetlands.

Sediment accretion rate and thus carbon sequestration rate decreased between 2013–2014 and 2019 at all four sites (Table 3). Since there was no statistical difference in carbon sequestration rates throughout the depth of each core at any site, it can be assumed that this decrease in carbon stored in the soils is due to a loss of carbon throughout the entire soil profile. Decreases in both sediment accretion rate and carbon sequestration could further point to potential peat collapse at these sites [26]. However, continued studies are needed to determine whether the peat collapse is due to hurricane activity or other processes.

### 3.5. Limitations

Limitations in this study include small sample size, spatial heterogeneity of carbon and roots across the soils, and temporal spacing between the two studies. At each site, only two soil cores were collected which does not gather the variability of the area. Since the soil cores were large (35.25 cm^2^), we were able to capture significant variability across the landscape as well as sample larger roots that would not have been collected in smaller cores, but future studies of the area should take additional samples to collect more of the spatial variability of carbon and roots across the landscape. With such a 5 year gap in time between sampling periods, there is a chance that other processes or minor disturbances played an effect on the carbon dynamics within the tidal creeks. However, as the only large disturbance within the time period, hurricane Irma is likely the cause of the reported changes. However, baseline samples should be collected more frequently so that they are able to capture pre-hurricane data with more accuracy and ensure that other variables are not causing the differences reported.

## 4. Materials and Methods

### 4.1. Study Site

**Figure 8:**
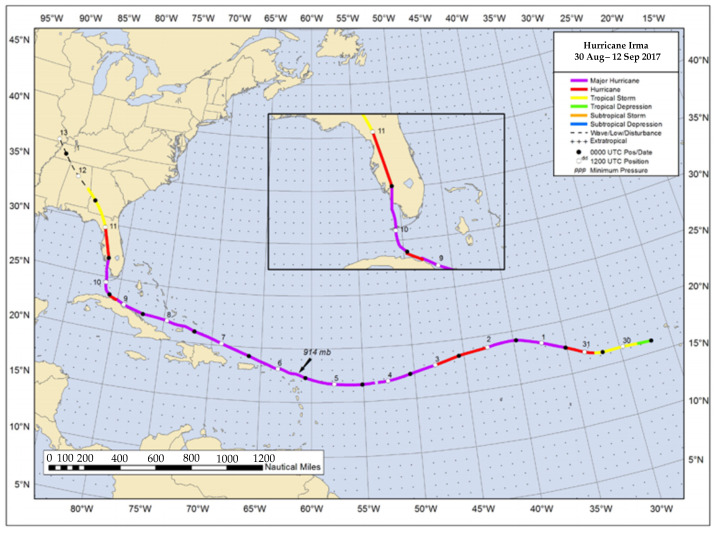
Track of Hurricane Irma through the Atlantic Ocean, Caribbean, and Florida from 30 August to 12 September 2017. The inset shows the path of Hurricane Irma as it hit Florida on 10 September 2017. From National Hurricane Center Tropical Cyclone Report [32].

**Figure 9:**
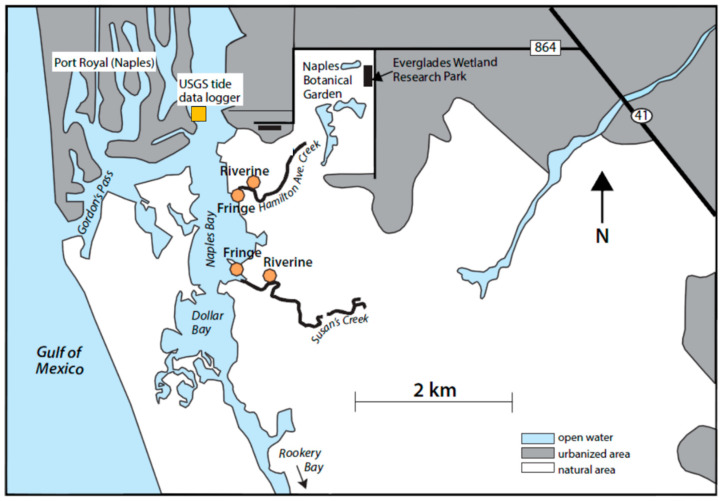
Sample sites on disturbed tidal creek (Hamilton Avenue Creek) and reference tidal creek (Susan’s Creek) in Naples Bay, Florida that were previously sampled by Marchio et al. [10] in the period 2013–2014 for carbon content and sequestration and were resampled in 2019 (Illustration from [10]).

In September 2017, the western coastline of Florida was directly hit by hurricane Irma (Figure 8). Hurricane Irma was, at the time, the strongest hurricane ever observed in the open Atlantic Ocean and hit Marco Island and Naples, Florida as a Saffir–Simpson category 3 major hurricane causing over $50 billion in damages in the United States (Cangialosi et al., 2018). This study analyzes the effect that hurricane Irma had on Naples Bay, Florida (25°5′ N, 81°47′ W) mangrove wetlands by comparing carbon concentrations that were analyzed in 4 soil core sampling sites pre-hurricane by Marchio et al. (2016) in July 2013 and January 2014 (pre-hurricane) to those collected at the same 4 sites in September 2019 (post-hurricane; Figure 9) on the southwest coast of Florida. Naples Bay is a shallow estuary (<7 m) which experiences semi-diurnal tides with 0.7–1.2 m water level variation under normal conditions [10,11,30]. Average annual temperature is 23.6 °C and annual precipitation is 1346 mm yr^−1^, with 60–65% of that occurring from June to September [11,30,31].

The study sites are on two tidal creeks, one hydrologically disturbed (Hamilton Avenue Creek) and the other as a hydrologically undisturbed reference creek (Susan’s Creek). Hamilton Avenue Creek is hydrologically disturbed as a result of upstream land use changes, dredging, and human development [10]. This has led to the creek being hydrologically isolated from both freshwater and marine surficial flows [33]. Both the disturbed and undisturbed creeks have a similar annual salinity (28 and 30 ppt, respectively) and turbidity (9.9 and 9.8 NTUs, respectively), but the reference creek has greater seasonal variability in salinity compared to the disturbed creek since natural freshwater inputs decrease salinity in the undisturbed creek during the wet season, whereas, in the disturbed creek, upstream hydrologic disturbance leads to salinity values that are only dependent on tidal influx [11,34]. These sites were selected to make a direct comparison with pre-hurricane samples to determine the effect of hurricane Irma on the long-term carbon sequestration capabilities of mangrove wetlands.

**Figure 10:**
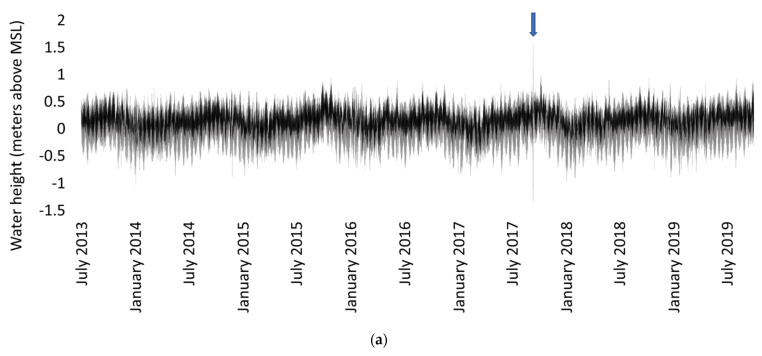

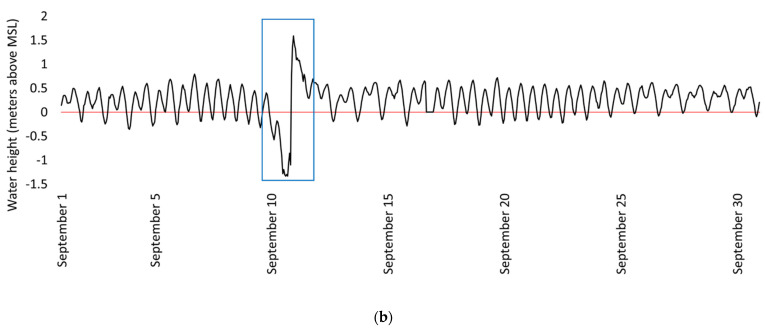
Water level in meters above mean sea level (MSL) from USGS station in the Gulf of Mexico on the Naples Pier (**a**) between July 2013 (pre-hurricane sampling) and July 2019 (post-hurricane sampling) with Hurricane Irma water level indicated by the arrow and (**b**) September 2017 water level with a box around hurricane Irma landfall.

Hurricane Irma caused a change in water level at the sites. A National Oceanic and Atmospheric Administration (NOAA) gauge in the Gulf of Mexico in Naples, approximately 5 km northwest of the study sites, captured water level throughout the hurricane and surrounding time period [35]. Typically, this station captures the semidiurnal tidal cycle with low tides at −0.3 to 0 m MSL and high tides at 0.5 to 0.75 m MSL during September 2017 (Figure 10) [35]. As a result of hurricane Irma, the water was initially pulled away from shore and water level was −0.5 m MSL at 02:12 GMT on 10 September 2017 and continued to decrease to the minimum water level of −1.3 m MSL at 17:06 GMT on 10 September 2017 (Figure 10) [35]. The water level then sharply increased to 1.6 m MSL at 22:36 GMT 10 September 2017 and stayed above the predicted water levels for approximately 36 h [35]. This was the only event with abnormal water levels recorded by the NOAA gauge between 2013 and 2019 (Figure 10a).

Although there is a 5.5 year gap between the initial sampling by Marchio et al. [10] and Cabezas et al. [11] and the post-hurricane samples collected in this study, there were no other major disturbances or irregular weather patterns. Hurricane Irma was the only major storm to make landfall in Naples, Florida since tropical storm Fay in 2008, more than 5 years prior to the original study [36]. Additionally, between the sampling dates there are no drought or freeze events on record for Naples, FL and only one above-average wet period in 2016 but there was no recorded coastal flooding as a result [36]. Tide gauge data (Figure 10a) indicate that between the sampling periods, the 24 h period surrounding hurricane Irma had the only abnormal water levels along the coast of Naples, Florida [35]. There were no significant land use or water source changes upstream of either tidal creek between 2014 and 2019. With no other major disturbances between sampling periods, it is likely that all major observed changes in carbon storage and sequestration rate between the samples are directly related to hurricane Irma and its corresponding hazards.

### 4.2. Sampling and Data Collection

Mangrove swamp soils in Naples Bay were collected at sites previously studied by Marchio et al. [10]. Two soil cores were sampled at each site from fringe and riverine hydrogeomorphic settings in the hydrologically disturbed creek and reference creek using a WaterMark universal core head sediment sampler (Forestry Suppliers Inc., Jackson, MS, USA) and 6.7 cm diameter polycarbonate-coring barrels. The barrels were inserted into the soil until rejection. If compaction took place during sampling, the core was discarded and repeated. The length of each core (ranging from 22 to 36 cm based on rejection point) was documented before being capped and sealed at both ends and stored upright at 4 °C to prevent core disturbance or carbon transformation. Additionally, one 5 m × 5 m plot was established at each site for mangrove forest community structure determination. One plot was established at each site surrounding the core sample sites to determine forest community structure at the sites where soil cores were collected. These sites were established by Marchio et al., and studied in the same manner [10]. Species present, diameter at breast height (DBH), and stem count were recorded to compare the mangrove forest community structure in 2019 to that described by Marchio et al. [10] and Cabezas et al. [11] in June 2014.

### 4.3. Lab Work

The soil cores were brought back to the Everglades Wetland Research Park (EWRP) lab at FGCU’s Kapnick Center located at Naples Botanical Garden where they were stored at 4 °C until the soil column was removed from the barrel, divided into 2 cm sections, and dried at 60 °C until weight was constant. Dry weight was recorded for all 2 cm sections and soil samples were ground and homogenized until the sample would run through a 2 mm sieve; roots larger than 2 mm were excluded from analytics. Bulk density was determined by using the recorded dry soil mass (M_d_) and original soil volume (V) in the following equation:

Bulk density = M_d_/V (1)

Using the DBH for each species of mangrove found in the reference and disturbed creeks, aboveground biomass (W_top_; including prop roots for *Rhizophera*) was determined using allometric equations developed for each species by Imbert and Rollet [37] and tested by Komiyama et al. [38] at various DBH intervals:

*Rhizophera mangle* L.    W_top_ = 0.178DBH^2.47^ (2)

*Avicennia germinans* (L.) L.    W_top_ = 0.0942DBH^2.54^ (3)

*Laguncularia racemosa* (L.) CF. Gaertin    W_top_ = 0.209DBH^2.24^ (4)

Carbon content was determined for each 2 cm segment. Two 50 mg samples of each section were analyzed for total carbon and inorganic carbon with a Shimadzu Total Organic Carbon Analyzer (TOC-L series, SSM-5000A, Kyoto, Japan). Percent total carbon was determined by combustion of the sample at 900 °C. Percent inorganic carbon was determined by first pre-treating the sample with 10 mol L^−1^ H_3_PO_4_ followed by combustion at 200 °C. Total organic carbon was calculated from the difference between total carbon and inorganic carbon content. Naples Bay carbon pools were compared to the similar data from pre-Irma sampling [10] and measured by the same instrument.

A high-efficiency Canberra germanium radiometric detector (GL 2820, Canberra, Australia) was used to measure ^210^Pb using the same instrument and methodology as Marchio et al. [10]. Ten-gram composite subsamples were created for each site, each was sealed in a glass, airtight tube with a rubber stopper. All oxygen was removed from the tube using a syringe. Each composite subsample was analyzed using gamma spectroscopy for 20 h and were read at 46.5 keV for ^210^Pb activity. Unsupported or excess ^210^Pb (the component of ^210^Pb that was deposited from the atmosphere as opposed to supported ^210^Pb which originates from in situ decay of ^226^Ra within the soil) was used to estimate the time of sediment deposition. Unsupported ^210^Pb was determined as the difference between the ^210^Pb activity at each depth and the ^210^Pb at the bottom of the core once it reached equilibrium (supported ^210^Pb). The constant rate of supply (CRS) model as defined in Equation (5) was used to calculate sediment age [39].

A = A_o_*e*^−kt^ (5)

where A is the unsupported ^210^Pb below the segment being dated, A_o_ is the total unsupported ^210^Pb in the soil column, k is the decay constant of ^210^Pb (0.0311 yr^−1^), and t is time (years).

Carbon sequestration was calculated for each depth by using Equation (6):

(6) $$C\;\mathrm{sequestration}\;=\;\frac{\left[C\right]\;\times \;M}{dt}$$

where [*C*] is the concentration of carbon at that depth (g-C kg^−1^), *M* is the mass of soil at that depth (kg m^−2^), and *dt* is the change in time from the previous depth (yr).

### 4.4. Statistical Analysis

Data were analyzed using JMP Pro 14 (SAS Institute Inc., Cary, NC, USA). The normality of the data was determined using the Shapiro–Wilk test. If the data met the assumptions for analysis of variance, a repeated-measures analysis of variance with a random effects-mixed model was used to determine difference in conditions including hydrogeomorphic settings and hydrologic disturbances within and between the two sampling periods. When the assumptions were not met and data could not be transformed to meet the assumptions, non-parametric Kruskal–Wallis analysis was performed to determine statistical differences in conditions between the sampling periods. A *p*-value of 0.05 was used to determine statistical significance.

## 5. Conclusions

This study analyzed carbon dynamics in mangrove soils and aboveground biomass in southwest Florida to help determine the effect of a singular Saffir–Simpson category 3 hurricane on carbon sequestration. We conclude the following:

- Mangroves are resilient and within two years of a hurricane, aboveground biomass production rebounds.
- Hurricanes cause a decrease in carbon in belowground stores. Aboveground carbon storage and biomass regeneration is important to buffer the overall carbon loss in mangroves.
- Due to carbon loss, post-hurricane mangroves may be a lower sink of carbon from the atmosphere for some number of years, thereby providing a positive feedback effect on climate change.
- With increased intensity of storms predicted in the tropics and subtropics due to climate change and with climate models projecting a steady increase in carbon dioxide in the atmosphere (1 percent per year) and tropical ocean surface temperatures rising by more than 2 °C by the end of the century [4], mangrove swamps are needed more than ever to provide a carbon sink while being resilient enough to continue to store carbon quickly after they are disturbed. It will be much more difficult, however, to restore mangroves to areas where humans have converted these natural ecosystems and their tidal creek watersheds. Current mangrove swamps must be protected so that they can continue to store carbon, protect humans from dangerous storms, and serve as nurseries for marine life.

## Figures and Tables

**Table 1:** Mangrove stem density and diameter at breast height (DBH) and estimated aboveground biomass of mangrove communities from 2013 to 2014 [10] and 2019 (this study) in hydrologically disturbed and reference creeks in Naples Bay, Florida ± standard error (number of samples).

			Hydrologically Disturbed Creek		Reference Creek	
			Riverine	Fringe	Riverine	Fringe
Tree density (stems m^−2^)	*R. mangle*	2013–2014	0.77	0.46	0.51	0.39
		2019	0.56	0.48	0.48	0.76
	*A. germinans*	2013–2014	-	-	-	0.01
		2019	-	-	-	-
	*L. racemosa*	2013–2014	0.21	0.18	0.01	0.03
		2019	0.48	0.04	-	0.08
Mean DBH (cm) ± SE	*R. mangle*	2013–2014	3.3 ± 0.2 (32)	3.2 ± 0.5 (38)	5.9 ± 0.8 (52)	5.5 ± 0.7 (39)
		2019	5.9 ± 0.7 (14)	5.0 ± 1.1 (12)	6.4 ± 0.5 (12)	5.5 ± 0.5 (19)
	*A. germinans*	2013–2014	-	-	-	24.4 (1)
		2019	-	-	-	-
	*L. racemosa*	2013–2014	7.0 ± 0.4 (10)	1.4 ± 0.2 (8)	25 (1)	18.2 ± 1.1 (3)
		2019	6.4 ± 0.9 (12)	0.9 (1)	-	12.4 ± 3.7 (2)
Aboveground biomass (kg m^−2^)	*R. mangle*	2013–2014	3.55 ± 0.02 (32)	6.01 ± 0.05 (38)	8.66 ± 0.02 (52)	5.39 ± 0.02 (39)
		2019	10.48 ± 0.21 (14)	9.18 ± 0.29 (12)	9.34 ± 0.15 (12)	11.46 ± 0.14 (19)
	*A. germinans*	2013–2014	-	-	-	3.13 (1)
		2019	-	-	-	-
	*L. racemosa*	2013–2014	4.88 ± 0.09 (10)	0.12 ± 0.01 (8)	2.83 (1)	4.55 ± 0.61 (3)
		2019	8.28 ± 0.21 (12)	0.01 (1)	-	5.23 ± 1.55 (2)
	Total	2013–2014	8.43 ± 0.03 (42)	6.14 ± 0.04 (46)	11.49 ± 0.05 (53)	13.08 ± 0.09 (43)
		2019	18.77 ± 0.15 (26)	9.18 ± 0.27 (21)	9.34 ± 0.15 (12)	16.69 ± 0.21 (21)

**Table 2:** Carbon gained (+) or lost (−) between 2013–2014 and 2019 sampling in the top 20 cm of soil in the disturbed and reference creeks.

Site	Inorganic Carbon Change (g-C m^−2^)	Organic Carbon Change (g-C m^−2^)
Disturbed Riverine	+0.21	−4071
Disturbed Fringe	−77	−706
Reference Riverine	+315	−433
Reference Fringe	−5.23	−5854
Average	+58	−2766

**Table 3:** Sediment accretion rate and carbon sequestration rate in 2013–2014 [10] and 2019 (this study) in the disturbed and reference creeks.

Site	Sediment Accretion Rate (mm yr^−1^)		Carbon Sequestration Rate (g-C m^−2^ yr^−1^)	
	2013–2014	2019	2013–2014	2019
Disturbed Riverine	3.04	1.91	126	44.9
Disturbed Fringe	2.23	1.79	74	62.6
Reference Riverine	5.43	4.02	162	154
Reference Fringe	2.28	3.69	127	70.0
Average	3.25 ± 0.75	2.85 ± 0.58	122.3 ± 18.1	82.9 ± 24.3

## Data Availability

The data presented in this study are available on request from the corresponding author.
